# Peristomal pyoderma gangrenosum: a review of nursing research progress

**DOI:** 10.3389/fmed.2026.1774931

**Published:** 2026-03-16

**Authors:** Guowei Chen, Xiaoxu Lan, Fang Li, Deyuan Zeng, Luhong Wang, Xiaoyu Chen, Li Zhu

**Affiliations:** 1 Department of General Surgery, Chongqing General Hospital, Chongqing, China; 2 Department of Hemodialysis, Chongqing General Hospital, Chongqing, China; 3 Department of Nursing, Xipeng Town Health Center, Chongqing, China

**Keywords:** immunosuppressive treatment, negative pressure wound therapy, ostomy complications, peristomal pyoderma gangrenosum, wound nursing

## Abstract

Peristomal pyoderma gangrenosum (PPG) is a rare and therapeutically challenging inflammatory dermatosis predominantly affecting individuals with stoma surgery. This review provides a comprehensive overview of PPG, encompassing its etiology, clinical manifestations, diagnostic methods, treatment strategies, and nursing management. PPG’s multifactorial etiology involves immune dysfunction, genetic predisposition, surgical trauma, and demographic factors, which contribute to the formation of painful ulcers that significantly impair patients’ quality of life. Accurate diagnosis requires a multidisciplinary approach, including clinical evaluation, histopathological examination, and advanced diagnostic criteria. Effective management combines local wound care, systemic therapies, and surgical interventions, emphasizing a patient-centered and evidence-based approach. Nursing strategies, including advanced wound care, pain management, psychological support, nutritional interventions, and health education, play a vital role in optimizing outcomes and preventing recurrence. Future research should focus on the development of specific biomarkers, novel therapeutic agents, and standardized nursing protocols to address the unmet needs in PPG care. This integrated diagnostic, therapeutic, and nursing framework aims to enhance patient outcomes and provide insights for future clinical and research advancements.

## 1 Introduction

Peristomal pyoderma gangrenosum (PPG) is a rare and highly morbid neutrophilic dermatosis characterized by rapidly progressive and painful ulceration of the peristomal skin. Although PPG is non-infectious, its clinical presentation often mimics common peristomal complications, including irritant contact dermatitis, fungal infections, and bacterial cellulitis, which may result in delayed diagnosis and inappropriate interventions. Notably, the phenomenon of pathergy—defined as an exaggerated cutaneous response to minor trauma—means that routine procedures, such as aggressive debridement, frequent appliance changes, or poorly fitting stoma systems, can precipitate lesion enlargement and exacerbate pain. Consequently, PPG is associated with prolonged wound duration, frequent healthcare utilization, increased treatment costs, and significant impairment in quality of life.

The management of PPG is complex and typically requires a combination of systemic immunomodulatory therapy and meticulous local wound and stoma care. In this context, nursing care is not merely supportive but plays a central role in achieving successful outcomes. Nurses often serve as the first point of contact for peristomal skin assessment, escalation of suspected atypical ulceration, and coordination of multidisciplinary care. Evidence-informed nursing interventions—such as atraumatic wound management, exudate control, pouching optimization, pain assessment and relief strategies, infection surveillance, nutritional support, psychological counseling, and structured patient education—directly influence healing trajectories and recurrence prevention. Meanwhile, heterogeneity in clinical presentation, the absence of universally accepted diagnostic pathways, and limited high-quality evidence specific to peristomal disease pose significant challenges to standardizing care.

In recent years, nursing research related to PPG has expanded from descriptive case-based experience to more structured approaches addressing assessment frameworks, multidisciplinary collaboration, patient-centered outcomes, and long-term self-management. However, gaps remain in early recognition algorithms, standardized nursing protocols tailored to different ulcer stages, best practices for appliance selection during active disease, and evaluation of education and psychosocial interventions.

Therefore, this review synthesizes current advances in nursing research and clinical practice related to PPG. We summarize etiologic and pathophysiologic insights, highlight key diagnostic considerations, outline contemporary treatment strategies, and emphasize practical, evidence-based nursing management across wound care, pain management, psychological support, nutrition, follow-up, and health education. By clarifying the nursing role within integrated care pathways, this review aims to facilitate earlier identification, safer peristomal management, improved patient experience, and more consistent clinical outcomes, while also identifying priorities for future research.

**Figure 1:**
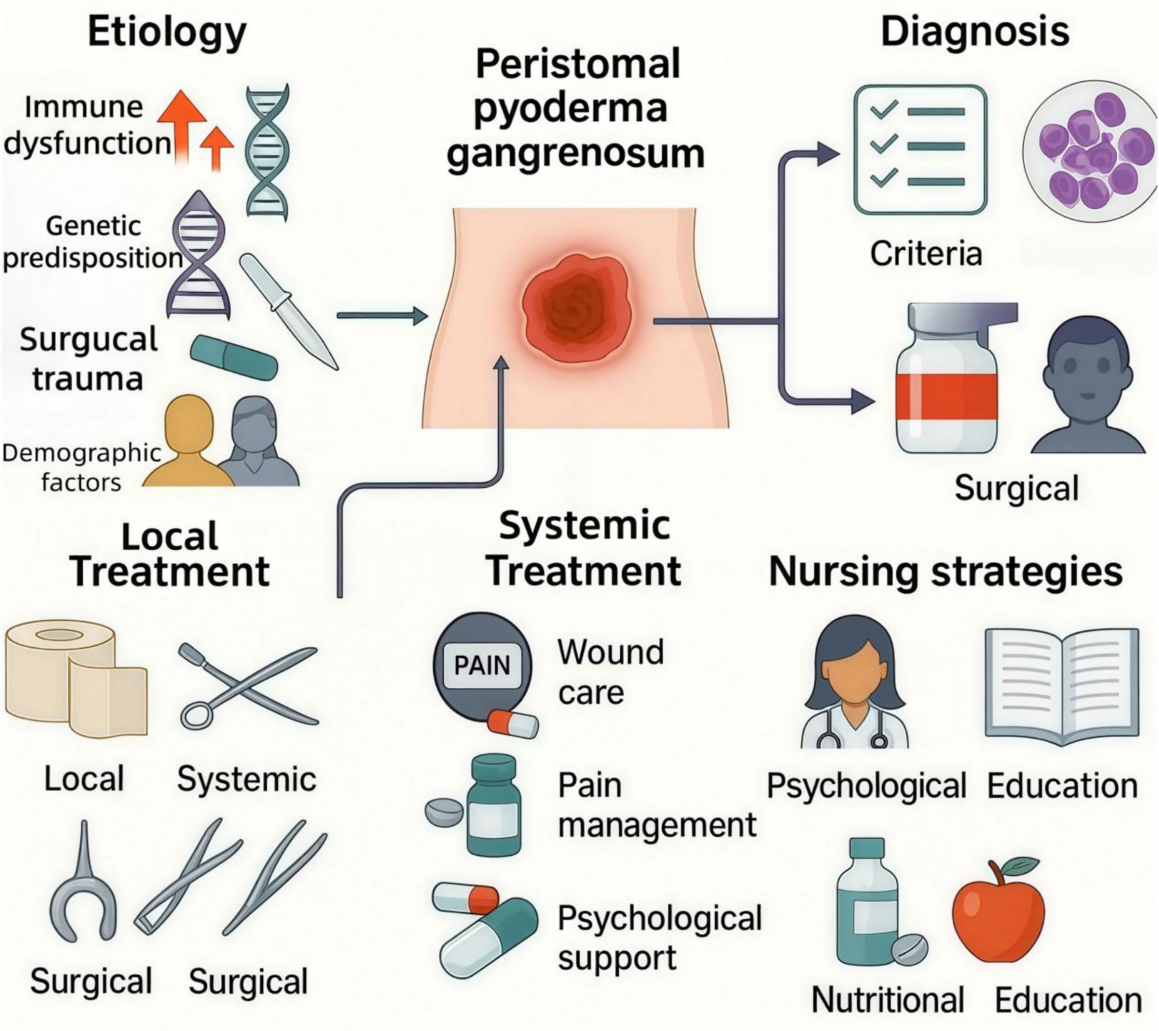
Integrated framework of peristomal pyoderma gangrenosum (PPG) management. Schematic overview summarizing major components of PPG care across the disease course. Etiology/pathogenesis includes immune dysfunction, genetic predisposition, surgical/traumatic triggers related to stoma creation and appliance-related microtrauma, and demographic or host factors. Diagnosis is based on a comprehensive clinical assessment supported by histopathology/exclusion of mimickers and application of established diagnostic criteria. Treatment is multimodal, combining local therapy (wound dressings and topical agents), systemic therapy (immunosuppressive/anti-inflammatory agents and biologics when indicated), and surgical approaches (selected procedures such as stoma revision/closure in refractory cases). Nursing strategies span evidence-based wound care, pain assessment and management, psychological support, nutritional support, and structured patient education to improve adherence and reduce recurrence. PPG, peristomal pyoderma gangrenosum.

To provide an overview of the key content areas discussed in this review, Figure 1 presents the integrated framework of peristomal pyoderma gangrenosum (PPG), encompassing etiologic contributors, diagnostic pathways, multimodal treatment approaches, and core nursing management strategies.

## 2 Etiology and pathogenesis of PPG

Peristomal pyoderma gangrenosum (PPG) accounts for approximately 15% of all cases of pyoderma gangrenosum (PG) (1–3). PPG presents a significant clinical challenge due to its rarity and complex pathology, sharing many features with typical PG, such as difficulty in identifying pathogens in cultures and non-specific inflammatory histological findings. Dense neutrophilic infiltration of the dermis is a pathological hallmark of PPG, consistent with its classification as a neutrophilic dermatosis. Despite ongoing research, the exact etiology of PPG remains elusive, with evidence pointing to multifactorial origins. Immune dysfunction, genetic predisposition, neutrophil abnormalities, and mechanical factors related to stoma appliances are suspected contributors. Notably, a history of trauma or surgery has been frequently documented in PPG cases, suggesting these events may serve as initiating factors. Although the incidence of PPG is relatively low, its impact is profound, particularly among patients with inflammatory bowel disease (IBD), who often endure chronic and recurrent episodes, severely affecting their quality of life.

### 2.1 Immunological factors

Immunological dysfunction plays a central role in the pathogenesis of PPG. This condition is strongly associated with systemic inflammatory diseases, particularly IBD (e.g., Crohn’s disease and ulcerative colitis), rheumatoid arthritis, and malignancies. Lyon et al. (4) reported that approximately 50% of PPG cases occur in individuals with systemic inflammatory diseases, underscoring the importance of immune-mediated mechanisms in disease development.

Further supporting this association, Cao (5) observed that PPG, while rare, is often an indicator of active disease in IBD patients. The heightened systemic inflammatory response driven by intestinal inflammation is believed to extend to cutaneous tissues, contributing to the formation of PPG lesions. Elevated levels of pro-inflammatory cytokines, such as tumor necrosis factor-alpha (TNF-α) and interleukins, have been implicated in promoting neutrophil activation and sustaining PPG progression.

**Figure 2:**
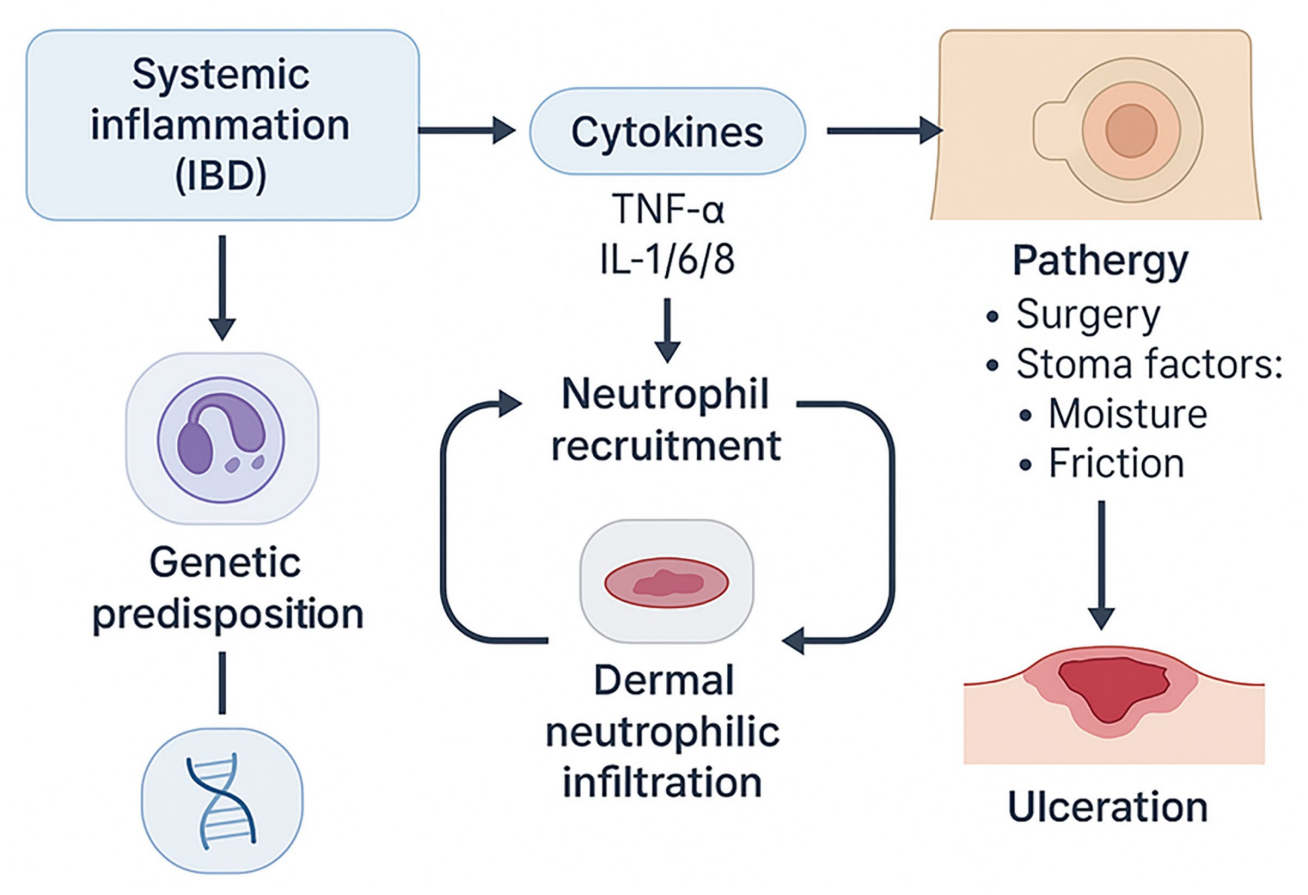
Immuno-mechanical pathogenesis of peristomal pyoderma gangrenosum (PPG). Schematic depiction of how systemic inflammation—particularly in inflammatory bowel disease (IBD)—promotes a cytokine milieu (e.g., TNF-α and interleukins) that drives neutrophil recruitment and dermal neutrophilic infiltration, contributing to tissue injury. Genetic predisposition may increase host susceptibility to dysregulated innate immune activation. At the stoma site, pathergy triggered by surgery and local factors (moisture, friction, and appliance-related microtrauma) further amplifies inflammation, ultimately resulting in rapid, painful ulceration typical of PPG. PPG, peristomal pyoderma gangrenosum.

Moreover, recent advances in biologic therapies, including TNF-α inhibitors and IL-12/23 inhibitors, have provided indirect evidence for the key immune pathways involved in PPG. Although primarily designed to treat systemic inflammatory diseases, these therapies have demonstrated efficacy in alleviating PPG lesions, further underscoring the link between systemic inflammation and localized neutrophilic dermatoses. Timely recognition and management of these immune triggers are paramount, as delays in diagnosis often result in worsened prognosis and increased recurrence rates. Collectively, IBD-associated systemic inflammation, cytokine dysregulation, and downstream neutrophil recruitment—together with host susceptibility and pathergy at the stoma site—constitute the immuno-mechanical basis of PPG ulceration (Figure 2).

### 2.2 Surgical factors

Surgical trauma is a well-recognized precipitating factor for PPG. PPG most commonly develops at stoma sites, where normal skin integrity is inherently disrupted. The physical disruption caused by surgery may initiate an exaggerated inflammatory response, particularly in patients with underlying immune dysregulation. Imaizumi et al. (6) highlighted that the inflammatory cascade initiated by surgical trauma can amplify the recruitment of neutrophils to the affected area, thereby exacerbating tissue damage and ulcer formation (7).

Beyond surgical trauma, stoma-specific environmental factors—such as fecal irritation, increased local moisture, friction, and sustained pressure—also contribute to PPG development. Frequent stoma appliance changes may further aggravate mechanical irritation, increasing the risk of repetitive microtrauma and subsequent inflammation (8). Clinical observations suggest that stoma appliances with poor fitting or inadequate skin protection exacerbate the condition. This underscores the need for meticulous stoma care and patient education to minimize these risks.

Recent innovations in stoma appliance design, particularly hydrocolloid-based skin barriers, have shown promise in reducing stoma-related skin complications. However, larger-scale studies are needed to validate their effectiveness in preventing PPG in high-risk populations.

### 2.3 Other related factors

Several demographic and genetic factors have been linked to PPG. Gender differences in PPG incidence have been noted, with international studies suggesting a higher prevalence among females. This disparity is hypothesized to be influenced by hormonal differences, as sex hormones are known to modulate immune responses and neutrophil activity (9). In contrast, studies conducted in China have reported a higher incidence among males, which may reflect sample size limitations or population-specific genetic and environmental factors.

Obesity has also emerged as a potential risk factor for PPG. While no direct causal relationship has been established, studies indicate that obese individuals exhibit elevated levels of pro-inflammatory chemokines and adipokines, which may predispose them to inflammatory conditions, including PPG (10). A higher body mass index (BMI) has been consistently observed among patients with PPG, suggesting that weight management may play a role in mitigating disease risk.

Additionally, genetic predisposition has been implicated in rare familial cases of PG, indicating a possible hereditary component in selected populations. Advances in genomic studies have begun to identify potential susceptibility loci associated with neutrophil function and inflammatory regulation, although further research is required to translate these findings into clinical practice.

## 3 Clinical manifestations and diagnosis

### 3.1 Clinical manifestations

Peristomal pyoderma gangrenosum (PPG) is a rare, non-infectious neutrophilic dermatosis characterized by destructive and painful peristomal skin ulcers that markedly impair quality of life. Clinical manifestations of PPG are heterogeneous and often begin as erythematous lesions that rapidly progress to vesicles, pustules, and ultimately deep ulcers. These ulcers typically exhibit undermined borders with a violaceous or purplish-red hue and are frequently accompanied by severe pain and persistent exudation (10). Localized around the stoma, these ulcers may progressively enlarge in depth and extent and, in severe cases, lead to stoma-site stenosis or necrosis.

The typical progression involves the transformation of erythematous plaques into painful ulcers with purulent discharge. Surrounding skin frequently displays erythema and inflammatory reactions, contributing to significant discomfort (1). Jiang (10) emphasized that the severe pain and excessive exudation associated with PPG ulcers frequently interfere with daily activities, including routine stoma care, thereby further complicating disease management.

Secondary infection of PPG ulcers may exacerbate symptoms, delay wound healing, and further complicate differentiation from other peristomal conditions. Given the aggressive and rapidly progressive nature of PPG lesions, prompt recognition and early intervention are essential to prevent rapid deterioration. Recent studies have highlighted that pain, exudation, and progressive ulceration are critical warning signs warranting immediate attention to distinguish PPG from other stoma-related complications, such as irritant dermatitis or fungal infections (11, 12).

### 3.2 Diagnosis

Diagnosing pyoderma gangrenosum (PG), including its peristomal variant, remains challenging due to the absence of specific biomarkers or pathognomonic clinical signs. Misdiagnosis is common, given the clinical overlap with other ulcerative and inflammatory conditions. To address this, several diagnostic criteria have been developed to improve diagnostic accuracy. The Su et al. criteria (13) and the Delphi International Consensus criteria (2018) (14) specifically target ulcerative PG, emphasizing clinical features, histological findings, and response to treatment.

The PARACELSUS diagnostic criteria, introduced by Jockenhöfer et al. (15), provide a novel scoring system that integrates clinical, histological, and treatment-related factors to achieve higher diagnostic specificity. This system has demonstrated significant utility in distinguishing PG from other conditions, such as infectious ulcers and vasculitic lesions, which often present with similar features.

From a practical perspective, these diagnostic frameworks differ primarily in their clinical use scenarios. The Su et al. (13) and Delphi criteria (14) are often applied in the early diagnostic phase to support clinical suspicion of ulcerative PG, particularly when classic features are present. In contrast, the PARACELSUS score (15) is especially valuable in diagnostically ambiguous cases, as its weighted scoring system enhances specificity and assists clinicians in differentiating PG from infectious or vasculitic ulcers. Awareness of these functional differences allows clinicians to select the most appropriate diagnostic approach based on disease presentation and diagnostic uncertainty.

Emerging diagnostic tools are exploring the potential of biomarkers, such as elevated neutrophil counts, inflammatory cytokines (e.g., TNF-α, IL-1, and IL-8), and genetic markers associated with autoinflammatory syndromes. However, their clinical application remains in its infancy (16). Imaging modalities, including high-resolution ultrasound and advanced wound imaging techniques, have also been proposed as adjunctive tools for assessing lesion depth and distinguishing PG from other ulcerative dermatoses (17).

In clinical practice, an integrative diagnostic approach remains the standard. This involves thorough documentation of the patient’s medical history, detailed clinical examination, histopathological evaluation of ulcer biopsies, and an assessment of treatment response. Particular attention should be given to ruling out conditions that mimic PPG, such as peristomal contact dermatitis, fungal infections, or Crohn’s disease-related cutaneous manifestations. Misdiagnosis not only delays appropriate treatment but may also exacerbate the disease through improper interventions, such as aggressive debridement, which can worsen PPG lesions (18, 19).

Ongoing research into non-invasive diagnostic techniques, including metabolomic and proteomic profiling, holds promise for improving diagnostic accuracy and early detection. Enhanced diagnostic tools, combined with education for clinicians on recognizing PPG, are essential for optimizing patient outcomes.

## 4 Treatment methods

**Figure 3:**
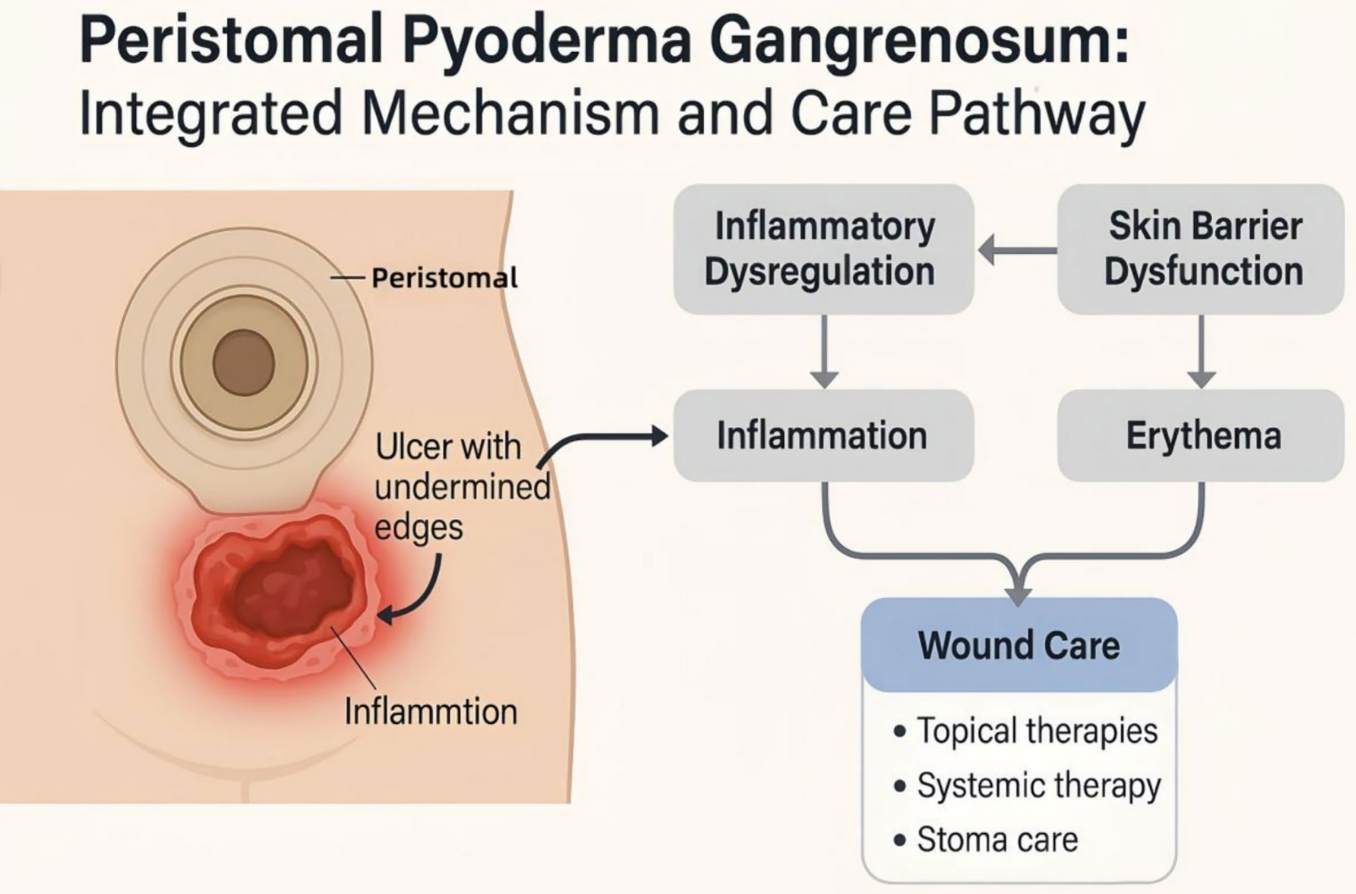
Peristomal pyoderma gangrenosum (PPG): integrated mechanism and care pathway. Conceptual diagram showing how inflammatory dysregulation and skin-barrier dysfunction contribute to peristomal erythema and inflammation, progressing to a characteristic painful ulcer with undermined edges. The figure also summarizes a practical care pathway emphasizing wound care as an integrated package that includes topical/local therapies (e.g., anti-inflammatory or antimicrobial topical approaches and appropriate dressings), systemic therapy targeting the underlying inflammatory process when indicated, and stoma care measures to reduce leakage, moisture, friction, and other peristomal triggers. PPG, peristomal pyoderma gangrenosum.

The management of peristomal pyoderma gangrenosum (PPG) requires a comprehensive and individualized approach, integrating local wound care, systemic therapies, and surgical interventions (20, 21). These strategies aim to address both the external manifestations and underlying pathological processes of PPG, thereby promoting faster recovery and minimizing recurrence risks. Effective treatment relies on a multidisciplinary collaboration among dermatologists (22), gastroenterologists, wound care specialists, nutritionists, and psychologists to ensure holistic care. Figure 3 illustrates an integrated mechanism-to-care pathway in PPG, linking inflammatory dysregulation and skin-barrier impairment to ulcer evolution and highlighting core management components (topical therapy, systemic therapy, and stoma-focused wound care).

### 4.1 Local treatment

Local wound care is the cornerstone of PPG management and should be initiated early to reduce pain, control inflammation, protect peristomal skin, and establish a stable microenvironment conducive to granulation and epithelialization. Because the peristomal region is continuously exposed to moisture, effluent irritation, and adhesive-related trauma, local therapy in PPG pursues two parallel objectives: (1) ulcer bed management and (2) peristomal protection with pouching optimization, both of which must be performed in an atraumatic manner to avoid pathergy-related deterioration.

#### 4.1.1 Debridement

Although debridement is a standard component of wound care, its application in PPG requires particular caution to avoid exacerbating tissue injury due to pathergy—a phenomenon where minor trauma worsens the condition. During the inflammatory or active stage, aggressive sharp debridement is generally avoided, and the priority is to remove loose devitalized tissue and reduce bioburden with the least possible mechanical injury. Selective debridement techniques, such as enzymatic or autolytic methods, are preferred to minimize tissue trauma (8). When debridement is necessary, clinicians should emphasize gentle irrigation, non-adherent contact layers, and minimal manipulation, and reassess frequently because an enlarging ulcer after a procedure may reflect pathergy rather than inadequate wound care.

#### 4.1.2 Wet dressings and dressing selection

Maintaining a moist wound environment accelerates healing by supporting autolysis, reducing desiccation-related pain, and promoting granulation. In practice, dressing selection should be individualized according to exudate level, undermining, and peristomal adhesive demands. Hydrocolloids, alginates, or hydrofibers are commonly employed to absorb exudate and maintain appropriate moisture levels (10). For highly painful ulcers, soft silicone contact layers or other non-adherent interfaces can reduce trauma during dressing removal. If undermining is present, gentle, loose cavity filling (rather than tight packing) can help manage dead space while avoiding pressure-related injury. Because peristomal leakage and moisture can rapidly macerate the surrounding skin, barrier films, skin protectants, and appropriately sized opening/cut-to-fit appliances are essential to maintain appliance adhesion and prevent repetitive skin stripping during frequent pouch changes.

#### 4.1.3 Negative pressure wound therapy

Also known as vacuum-sealed drainage (VSD), negative pressure wound therapy (NPWT) reduces edema, improves local perfusion, supports angiogenesis, and enhances granulation tissue formation. Its role in PPG treatment is promising, particularly for large or non-healing ulcers, as demonstrated in Bazaliński’s et al. (12) case studies. In clinical application, NPWT should be regarded as an adjunct rather than a substitute for anti-inflammatory control, and the technique should be modified to reduce procedure-related trauma (e.g., protective contact layer over the ulcer bed, careful foam placement, and gentle dressing change technique). For peristomal ulcers, NPWT can be especially valuable when exudate is heavy or wound size impairs pouch adhesion, but it requires close monitoring to avoid pain exacerbation or edge deterioration.

#### 4.1.4 Topical antibiotics and glucocorticoids

These agents play a critical role in controlling local infection and inflammation. Topical glucocorticoids, such as clobetasol, reduce neutrophilic infiltration and are often combined with antimicrobial therapies to manage secondary infections (22). Importantly, clinicians should distinguish colonization from clinically significant infection; when purulence, malodor, increasing erythema, or systemic signs suggest infection, targeted local antisepsis and culture-guided therapy may be needed, while unnecessary antibiotic exposure should be avoided. In localized lesions or as a bridge while systemic therapy is initiated, topical anti-inflammatory therapy can help reduce pain and halt peripheral expansion, especially when combined with atraumatic dressings and meticulous peristomal skin protection.

#### 4.1.5 Emerging/adjunctive local approaches

Recent innovations, such as the use of bioengineered skin substitutes, dermal matrices, and advanced wound dressings infused with antimicrobial agents, offer additional options for local management. Potential advantages include faster coverage of healthy granulation tissue, reduced dressing-change frequency, and improved control of bacterial burden in exudative wounds. However, because PPG is an immune-mediated condition with pathergy risk, these modalities are generally considered after inflammation is controlled and require further validation in PPG-specific studies before routine recommendation.

### 4.2 Systemic treatment

Systemic therapy is often required in PPG because the disease is driven by dysregulated innate immunity and neutrophil-mediated inflammation, and local measures alone may be insufficient when ulcers are rapidly progressive, highly painful, undermined, extensive, or refractory. In clinical practice, systemic treatment should be individualized according to disease severity, rate of progression, comorbidities (especially IBD and other inflammatory disorders), and patient risk factors (e.g., infection risk, diabetes, osteoporosis, renal dysfunction). The overarching goals of systemic therapy are to rapidly suppress active inflammation, prevent further pathorgic expansion, achieve durable healing, and minimize drug toxicity through timely tapering and steroid-sparing strategies.

#### 4.2.1 Glucocorticoids

Oral corticosteroids (e.g., prednisone/prednisolone) remain first-line systemic agents owing to their rapid anti-inflammatory effects and ability to quickly reduce pain and halt ulcer enlargement. They are commonly used as induction therapy, followed by gradual tapering once clinical stabilization and early granulation are achieved. However, long-term use is limited by adverse effects, including hyperglycemia, hypertension, gastrointestinal complications, osteoporosis, mood changes, and increased infection risk (1). Accordingly, preventive measures (e.g., bone protection strategies when appropriate), close monitoring, and early transition to steroid-sparing regimens are essential, especially in patients with prolonged courses or relapse tendency.

#### 4.2.2 Immunosuppressants (steroid-sparing agents)

For patients with refractory disease, contraindications to high-dose steroids, or the need for long-term control, immunosuppressive agents are frequently used either as monotherapy or in combination with glucocorticoids. Cyclosporine can provide relatively rapid disease control and is often considered alongside systemic steroids as a core option for active PG/PPG; however, clinicians must monitor for nephrotoxicity, hypertension, electrolyte disturbances, and drug–drug interactions. Mycophenolate mofetil and methotrexate are also used as steroid-sparing agents, particularly for maintenance or chronic control, though onset may be slower and requires ongoing monitoring for cytopenias, hepatic toxicity, and infection risk (8, 15). In nursing practice, structured monitoring (blood pressure, renal function, liver enzymes, blood counts) and patient counseling on infection warning signs and adherence are critical to safely sustain systemic immunosuppression.

#### 4.2.3 Biologic therapies

Targeted biologics (23, 24) have become increasingly important for neutrophilic dermatoses, particularly when conventional agents fail, toxicity limits escalation, or inflammatory comorbidities provide a strong mechanistic rationale for cytokine blockade. TNF-α inhibitors (e.g., infliximab, adalimumab) are among the most commonly used biologics in PG/PPG and may be particularly effective in patients with concomitant IBD, where they can simultaneously control intestinal inflammation and cutaneous ulcer activity (18). IL-12/23 inhibition (e.g., ustekinumab) has also shown benefit in selected refractory cases, again with particular relevance to IBD-associated disease. When biologic therapy is initiated, baseline screening and ongoing surveillance (e.g., latent infection risk and treatment-related adverse events) are essential, and coordination among dermatology, gastroenterology, surgery, and nursing teams can improve safety and continuity of care (25).

#### 4.2.4 Traditional Chinese medicine

In some settings, traditional Chinese medicine (TCM) is used as an adjunct to reduce systemic inflammatory burden and support wound healing, and herbal formulations those reported by Toyoda et al may offer symptom relief and supportive benefits. However, clinical translation is challenged by heterogeneity in formulations, variable dosing and preparation, and limited high-quality controlled evidence. Therefore, TCM should be considered as complementary rather than substitutive therapy, with careful attention to potential herb–drug interactions when combined with steroids, immunosuppressants, or biologics.

#### 4.2.5 Combination and monitoring strategy

Comprehensive systemic regimens commonly adopt an induction–maintenance approach: rapid control with glucocorticoids, early addition of steroid-sparing immunosuppressants or biologics for sustained control, and individualized tapering to reduce cumulative toxicity. Regular monitoring is essential to balance therapeutic benefits against adverse effects, with particular attention to infection risk, metabolic complications, renal/hepatic function, hematologic parameters, and patient-reported outcomes such as pain burden and quality of life. Multidisciplinary coordination and consistent follow-up further reduce inappropriate procedural triggers (e.g., traumatic interventions) and support adherence throughout prolonged healing courses.

### 4.3 Surgical interventions

Surgical interventions play an important role in managing complex or refractory cases of PPG, particularly when medical therapy alone fails to control disease progression or restore functional peristomal integrity. However, due to the phenomenon of pathergy—where surgical trauma may exacerbate existing ulcers or induce new lesions—surgical intervention must be carefully timed, cautiously performed, and coordinated within a multidisciplinary framework. Most surgical interventions in PPG are considered adjunctive to systemic immunosuppression, not standalone solutions.

#### 4.3.1 Debridement and flap reconstruction

For ulcers that are extensive, deep, or complicated by necrotic tissue and non-healing margins, surgical debridement may be required to remove devitalized tissue and prepare the wound bed for closure or reconstruction. However, unlike typical chronic wounds, aggressive sharp debridement is generally discouraged during the early inflammatory phase of PPG, as it may exacerbate local tissue injury. Instead, debridement is typically deferred until adequate inflammatory control is achieved and the wound bed demonstrates healthy granulation. In selected cases, extensive tissue loss may necessitate reconstructive procedures such as local or regional flap transplantation, especially when the peristomal skin is extensively compromised, or pouching becomes impossible due to wound geometry (27). When flap coverage is pursued, ensuring adequate immunosuppressive control perioperatively is crucial to minimize flare-ups and graft failure.

#### 4.3.2 Stoma revision or closure

In cases where persistent mechanical irritation, leakage, or poor appliance fit contribute to ongoing pathergy and non-healing, stoma revision (e.g., relocation to a healthier site) may be necessary. This is particularly relevant in patients with unfavorable stoma positioning, retraction, or prolapse, where appliance sealing is inadequate. In extreme or refractory situations, permanent stoma closure may be considered, especially when fecal diversion is no longer essential. Imaizumi et al. (6) reported that complete stoma closure led to resolution of severe, unresponsive PPG lesions in IBD patients, likely due to the removal of chronic irritation and restoration of skin integrity (28). Such decisions require shared decision-making involving the patient, colorectal surgeon, dermatologist, and wound care nurse.

#### 4.3.3 Combined surgical–medical management

Optimal outcomes often rely on a multimodal approach, integrating surgical intervention with systemic therapy. Immunosuppressive control before and after surgery helps reduce the risk of surgical trauma triggering new or worsening ulcers. In high-risk or immunocompromised patients, perioperative planning should include wound management, nutritional optimization, infection prophylaxis, and individualized immunosuppressive tapering schedules. Nursing staff play a central role in this process by monitoring for early signs of recurrence, ensuring gentle dressing care, maintaining appliance integrity post-surgery, and coordinating follow-up across disciplines. In many institutions, management of such complex cases benefits from multidisciplinary team (MDT) rounds, where dermatology, surgery, stoma care, nursing, and internal medicine collaborate in real-time to balance risk and timing.

In summary, surgery is not routine in PPG but is essential for selected patients with extensive tissue damage, stoma-related mechanical failure, or refractory disease despite optimized medical therapy. Careful timing, immunologic suppression, and team-based coordination are prerequisites for safe and successful surgical outcomes. A concise summary of the major treatment modalities, categorized into local, systemic, and surgical approaches, is provided in Table 1 to support clinical decision-making.

**Table 1:** Treatment strategies for peristomal pyoderma gangrenosum (PPG).

Treatment category	Specific measures	Details and goals	References
Local treatment	Debridement	Removes necrotic tissue to control infection and promotes granulation tissue growth	Xu et al. (25)
	Moist wound dressing	Removes necrotic tissue to control infection and promotes granulation tissue growth	Baranoski et al. (17)
	Negative pressure wound therapy (NPWT)	Removes necrotic tissue to control infection and promotes granulation tissue growth	Bazaliński et al. (12)
	Topical antibiotics/glucocorticoids	Reduces local inflammation and bacterial colonization	Lyon et al. (4)
Systemic treatment	Immunosuppressants	Controls underlying systemic inflammation; commonly used agents include corticosteroids and calcineurin inhibitors	Jockenhöfer et al. (15)
	Biologic agents	Targets specific immune pathways, e.g., TNF-α inhibitors (infliximab) or IL-12/23 inhibitors (ustekinumab)	Chatzinasiou et al. (16)
	Traditional Chinese medicine (TCM)	Utilizes herbal formulations to address systemic inflammation; limited by variability in preparation and evidence	Yang et al. (26)
Surgical treatment	Flap transplantation	Provides coverage for large or non-healing ulcers by transferring tissue from a donor site	Becker et al. (27)
	Stoma revision/closure	Corrects mechanical factors contributing to peristomal skin complications, potentially resolving PPG in severe cases	Imaizumi et al. (6)
	Combined approaches	Requires multidisciplinary collaboration among dermatology, surgery, and gastroenterology teams for complex cases	Guo et al. (18)

Summary of key interventions used in the management of PPG, categorized into local, systemic, and surgical approaches. Each modality is paired with its therapeutic goal and supporting literature to aid in clinical interpretation and multidisciplinary planning.

### 4.4 Multidisciplinary collaboration and clinical monitoring

A multidisciplinary approach is essential for the effective management of PPG, given the complexity of its pathogenesis, variable clinical course, and high risk of misdiagnosis or suboptimal treatment. Effective care requires dynamic coordination among dermatologists, colorectal or general surgeons, wound care nurses, gastroenterologists (particularly in IBD-associated cases), nutritionists, pain specialists, and psychosocial support professionals. Each discipline contributes unique expertise that enables individualized, stage-specific, and patient-centered care—from accurate diagnosis and medical therapy to stoma management, psychological support, and long-term follow-up.

In most institutions, early referral to dermatology or wound care specialists improves diagnostic accuracy and reduces inappropriate interventions such as traumatic debridement or unnecessary antimicrobial use. Nurses with specialized wound and ostomy training (e.g., WOCN-certified) are essential for ongoing assessment, atraumatic dressing care, and optimizing pouching systems to minimize peristomal leakage and protect wound margins. Gastroenterologists play a central role in patients with comorbid IBD by adjusting immunosuppressive regimens or biologics to simultaneously control intestinal and cutaneous inflammation. Meanwhile, dietitians assess and correct nutritional deficiencies, particularly in protein, zinc, vitamin C, and other nutrients that impair healing, and recommend enteral or parenteral supplementation when needed.

Multidisciplinary rounds or case conferences can improve clinical decision-making by synchronizing goals across specialties, especially when determining the timing of surgery, escalation of immunosuppression, or transitions to home-based wound care. Equally important, nursing-led coordination helps ensure consistent monitoring and continuity of care across settings (inpatient, outpatient, home health).

#### 4.4.1 Clinical monitoring

Regular clinical assessment is key to evaluating treatment response and guiding timely modification of interventions. Essential components of wound monitoring include ulcer size (length, width, depth), edge morphology and undermining progression, exudate volume, color, and odor, granulation quality and epithelialization, pain severity and response to analgesia, and signs of local or systemic infection.

Documentation using validated wound assessment tools, wound photography (with patient consent), and structured monitoring protocols can enhance continuity and enable objective longitudinal comparison. In research settings or advanced wound centers, digital wound imaging systems provide quantitative metrics for tracking surface area and healing velocity.

#### 4.4.2 Emerging technologies

Advances in artificial intelligence (AI) and digital health tools offer new possibilities for precision wound monitoring. AI-based image analysis algorithms are being developed to classify wound types, predict healing trajectories, and detect early signs of complications. When integrated with electronic medical records (EMRs), these technologies may support real-time alerts for worsening ulcers or delayed healing, allowing earlier intervention. Mobile apps that enable remote wound assessment by nurses or patients—using smartphone cameras and cloud-based dashboards—may improve access to specialist guidance in remote areas or for patients with mobility limitations.

Although such technologies are still maturing, their integration into multidisciplinary care pathways could enhance efficiency, personalization, and early detection of treatment failure, particularly for chronic, complex wounds like PPG. Future studies should explore their utility specifically in peristomal contexts, where anatomical challenges and appliance interference can complicate assessment.

## 5 Nursing and management

**Figure 4:**
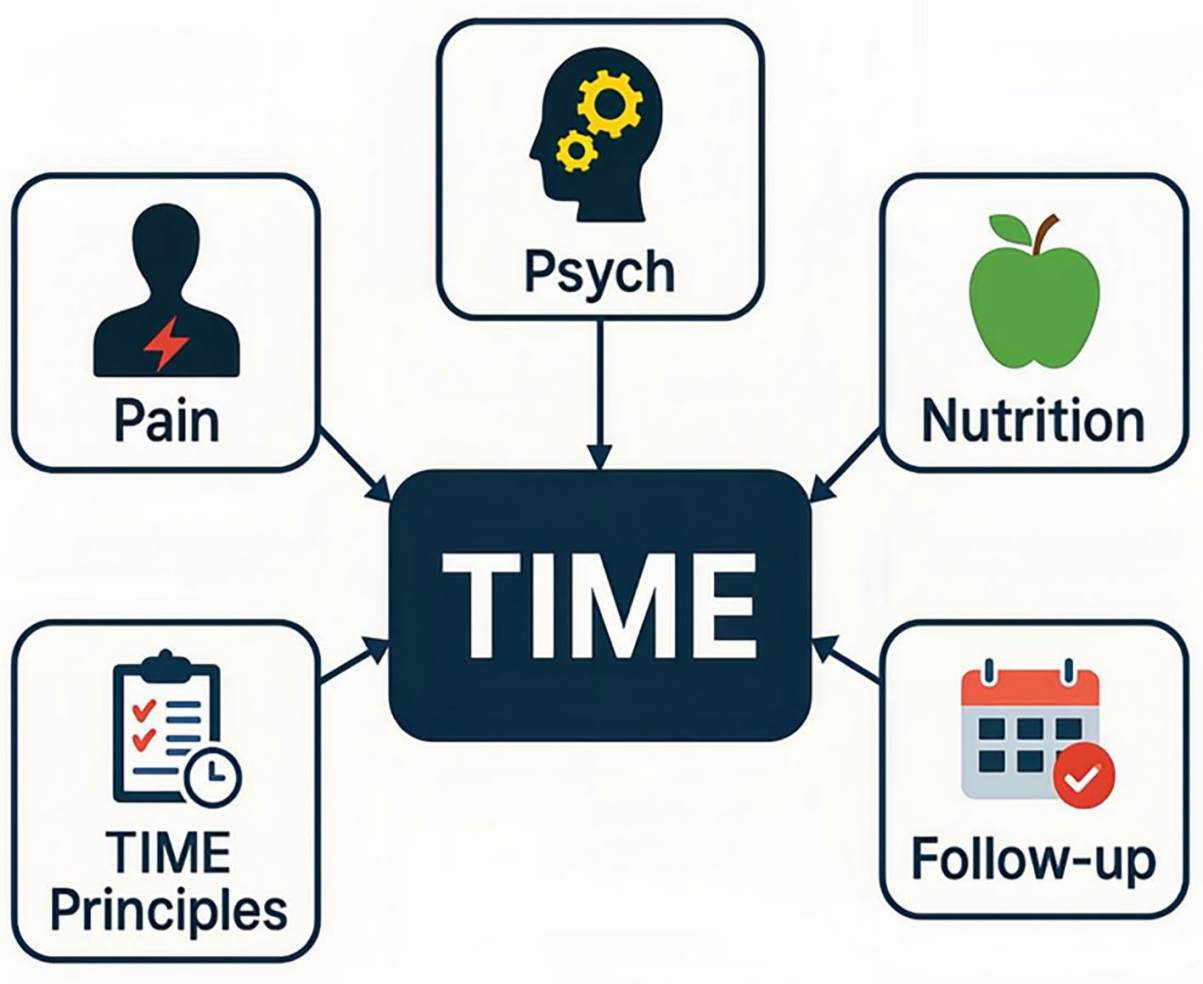
Nursing management framework for peristomal pyoderma gangrenosum (PPG). Framework highlighting a practical, holistic nursing pathway centered on TIME wound-bed preparation and coordinated supportive care. TIME includes tissue management (atraumatic removal of nonviable tissue while minimizing pathergy), infection/inflammation control, moisture balance (exudate management and protection of peristomal skin), and edge assessment (monitoring undermining/erythema progression and treatment response). Surrounding this core, nursing priorities include pain assessment and multimodal pain relief, psychological support (screening and counseling to reduce anxiety/depression and improve adherence), nutritional assessment and targeted supplementation, and planned follow-up with health education to optimize self-management, prevent recurrence, and improve quality of life. PPG, peristomal pyoderma gangrenosum.

Nursing strategies for peristomal pyoderma gangrenosum (PPG) constitute essential components of patient care, with the goals of improving healing outcomes, minimizing recurrence, and enhancing quality of life. Despite the limited number of specialized protocols globally, current nursing practices emphasize five key domains: wound care, pain management, psychological support, nutritional support, and health education. These strategies are rooted in evidence-based practices and tailored to meet the unique clinical challenges posed by PPG. To operationalize these domains in daily practice, we propose a nursing management framework centered on TIME-based wound principles and coordinated supportive care (pain, psychological health, nutrition, and structured follow-up/education) (Figure 4).

### 5.1 Wound care

Wound care is a cornerstone of nursing management in PPG and requires an individualized, atraumatic, and evidence-based approach that integrates clinical assessment, cleansing, dressing selection, moisture and exudate control, and protective ostomy care. The goal is to not only accelerate wound healing but also prevent further deterioration due to pathergy, minimize pain, and optimize peristomal appliance function.

#### 5.1.1 Wound assessment

Accurate and consistent wound assessment forms the foundation of effective wound care. Structured tools, such as the Bates-Jensen Wound Assessment Tool and the Wound, Ostomy and Continence Nurses (WOCN) Society’s Wound Assessment Guidelines, provide standardized methods for evaluating parameters including ulcer size, depth, edge quality, granulation tissue, necrosis, and exudate characteristics (29–33). These tools support both baseline assessment and longitudinal monitoring, enabling clinicians to detect subtle changes early and adjust care accordingly.

Advanced technologies such as 3D wound imaging, optical measurement systems, and high-frequency ultrasound have shown promise in improving wound documentation accuracy and facilitating volumetric tracking over time, especially in wounds with undermining or irregular topography (25–27, 34). These modalities can be especially useful in research settings or specialty clinics and may help distinguish between healing stagnation and progression.

#### 5.1.2 Wound care principles: the TIME framework

The internationally endorsed TIME framework (tissue management, infection/inflammation control, moisture balance, and edge advancement) provides a systematic structure for wound bed preparation and ongoing care:

1) Tissue management: Removal of non-viable or necrotic tissue is important for reducing bacterial load and promoting healthy granulation. However, in PPG, where pathergy is common, non-traumatic debridement methods are essential. Autolytic debridement using hydrogels, enzymatic agents, or moisture-retentive dressings is often preferred over sharp or surgical debridement, particularly during the active inflammatory phase (8). Mechanical trauma must be minimized, and frequent reassessment is crucial to avoid triggering ulcer expansion.
2) Infection control: PPG ulcers may be colonized or secondarily infected due to their location and chronicity. Antiseptic or antimicrobial dressings—such as silver-impregnated foams, iodine-based agents, or polyhexamethylene biguanide (PHMB)—can help reduce bacterial burden, especially in exudative wounds. Culture and sensitivity testing should guide topical or systemic antibiotic use when clinical signs of infection are present (e.g., increased purulence, odor, periwound erythema) (17).
3) Moisture balance: Maintaining a moist wound environment enhances autolysis, supports cellular migration, and reduces pain. At the same time, excess exudate must be controlled to prevent maceration of the surrounding skin and undermine pouch adhesion. Dressings such as alginates, hydrofibers, hydrocolloids, and foam dressings are commonly used to strike this balance. In highly exudative wounds, layered dressings with secondary absorption may be needed, and exudate should be monitored for color and volume changes indicative of infection or healing progression.
4) Edge assessment: Regular evaluation of wound edge morphology is vital for tracking progression or detecting early signs of deterioration. Advancing epithelial edges suggest healing, whereas expanding erythema, rolled or undermined edges, or increasing purulence may signal worsening inflammation, uncontrolled infection, or inadequate wound management (17). Edge care also guides dressing choice and frequency of change.

#### 5.1.3 Ostomy care integration

Effective ostomy care is inseparable from wound care in PPG. The peristomal skin must be protected against leakage, mechanical stripping, and friction—all of which can perpetuate trauma and delay healing. Use of anti-leakage systems (e.g., convex or flat-base plates, hydrocolloid barriers, and sealing rings) helps protect the wound margin and support longer pouch wear times. Protective barrier films or pastes can shield vulnerable skin from effluent exposure and adhesive injury (29, 32).

Patient and caregiver education plays a pivotal role in ostomy self-care. Teaching proper bag-emptying techniques, gentle appliance removal, skin inspection, and when to seek help can reduce complications and empower self-management. Toyoda et al. (26) reported that the use of flat-base appliances significantly reduced leakage in PPG patients by optimizing peristomal fit. Nurses should also assess for appliance compatibility with wound dressings and help patients adapt pouching techniques during different stages of healing.

### 5.2 Pain management

Pain is one of the most prominent and debilitating symptoms of PPG and is often more severe than that observed in other chronic peristomal wounds. Severe pain not only limits mobility, sleep, and daily activities but also adversely affects treatment adherence, wound care tolerance, and psychological well-being. Effective pain management is therefore a core nursing responsibility and should be implemented early, continuously reassessed, and integrated into the overall medical–surgical treatment plan.

#### 5.2.1 Pain assessment

Systematic pain assessment is essential for guiding individualized interventions and evaluating therapeutic effectiveness. Standardized self-report tools, such as the Numeric Rating Scale (NRS) and the Faces Pain Scale-Revised (FPS-R) allow quantification of pain intensity and facilitate communication across care teams (35). These tools are particularly useful for tracking changes before and after wound care procedures, dressing changes, or treatment escalation.

For patients who are unable to reliably self-report pain due to cognitive impairment, severe illness, or communication barriers, observational assessment tools, such as the Adult Pain Behavior Assessment Scale, are recommended to evaluate behavioral indicators, including facial expression, guarding, vocalization, and physiological responses (35). Beyond pain intensity, nurses should assess pain quality (burning, stabbing, throbbing), temporal patterns (constant vs. procedural pain), and triggers (dressing removal, leakage, movement), as these features may reflect underlying inflammatory activity or neuropathic components.

#### 5.2.2 Pain management strategies

Given the multifactorial nature of pain in PPG, optimal management relies on a multimodal approach that combines pharmacologic and non-pharmacologic interventions, coordinated across disciplines.

##### 5.2.2.1 Pharmacologic interventions

Pharmacologic pain management should be tailored according to pain severity, mechanism, and patient tolerance. Topical anesthetics may be applied before dressing changes to reduce procedural pain and improve patient cooperation. Systemic analgesics, such as acetaminophen or nonsteroidal anti-inflammatory drugs (NSAIDs), are commonly used for mild to moderate pain, provided there are no contraindications (27). In cases where pain exhibits neuropathic features—such as burning, shooting sensations, or allodynia—adjuvant agents, including gabapentinoids, may be beneficial (27).

Importantly, pain control should be closely synchronized with anti-inflammatory treatment, as inadequate suppression of disease activity may result in persistent or escalating pain despite escalating analgesics. Nurses play a critical role in monitoring analgesic efficacy, identifying adverse effects (e.g., gastrointestinal symptoms, sedation), and advocating for treatment adjustments when pain remains uncontrolled.

##### 5.2.2.2 Non-pharmacologic approaches

Non-pharmacologic strategies are valuable adjuncts that enhance comfort, reduce anxiety, and improve coping. Techniques such as relaxation training, guided imagery, and cognitive-behavioral therapy (CBT) can help patients manage pain perception and emotional distress associated with chronic wounds (14). Transcutaneous electrical nerve stimulation (TENS) has also been used to modulate pain signaling and may be particularly useful for patients seeking to minimize medication burden (14).

In addition, atraumatic wound care practices—including gentle cleansing, non-adherent dressings, and minimizing dressing change frequency—are essential nursing interventions that directly reduce pain intensity. Educating patients about pain expectations, self-monitoring, and when to request assistance can further enhance perceived control and adherence.

#### 5.2.3 Integrated pain management model

The integrated medical–nursing pain management model emphasizes close collaboration among nurses, physicians, pain specialists, and mental health professionals to develop individualized, adaptive pain control plans. Regular reassessment—particularly before and after wound care procedures—allows timely modification of strategies and prevents pain escalation. By addressing both physical and psychosocial dimensions of pain, this coordinated approach improves patient comfort, supports ongoing wound care, and enhances overall quality of life.

### 5.3 Psychological support

Peristomal pyoderma gangrenosum (PPG) imposes a substantial psychological burden due to its chronic course, pain, disfigurement, and frequent delays in diagnosis or mismanagement. Patients frequently experience anxiety, depression, social withdrawal, poor body image, and feelings of hopelessness, especially when ulceration recurs or fails to respond to treatment. These psychological sequelae are further amplified by visible skin damage, impaired mobility, loss of independence, and stigma related to ostomy care.

As a result, psychological support is not ancillary but integral to holistic nursing management in PPG. Early identification of emotional distress, timely referral for counseling, and structured mental health interventions can improve adherence, wound healing outcomes, and overall quality of life.

#### 5.3.1 Counseling and support groups

Professional psychological counseling is recommended for patients experiencing clinically significant depression, anxiety, or adjustment disorder related to chronic wound care. Mental health providers can help patients process trauma associated with visible disfigurement or pain and equip them with coping strategies to manage uncertainty about disease recurrence.

In parallel, peer support groups provide unique emotional benefits. These forums allow patients to share lived experiences, exchange practical tips for stoma care and daily function, and gain validation from others facing similar challenges. Participation in support groups has been shown to foster resilience, reduce isolation, and build hope, especially in patients with long-standing disease (18).

Importantly, nurses can facilitate these connections by identifying local or online support networks, providing referrals, or organizing structured group education sessions that include both medical and psychosocial components.

#### 5.3.2 Education and empowerment

Health literacy and emotional preparedness directly influence patient engagement and self-management capacity. Providing clear and empathetic education about the nature of PPG, its inflammatory pathogenesis, expected healing trajectory, and treatment goals can reduce uncertainty and fear. Patients who understand their condition are more likely to participate in shared decision-making and adhere to complex care routines, including ostomy management and systemic therapy.

Individualized counseling sessions led by nurses or multidisciplinary educators allow patients to voice personal concerns, clarify misconceptions, and receive tailored reassurance (35). This is especially crucial when addressing worries about recurrence, long-term immunosuppression, or surgical interventions. Education should also include family members or caregivers, who often play a central role in outpatient wound care and emotional support.

#### 5.3.3 Holistic care integration

Holistic nursing care for PPG integrates mind–body interventions designed to reduce stress, improve sleep, and enhance overall coping capacity. Strategies include:

Relaxation exercises, such as deep breathing or progressive muscle relaxation.
Mindfulness-based stress reduction (MBSR) techniques to reduce pain-related rumination.
Sleep hygiene education, including establishing consistent sleep routines and minimizing stimulants before bed.
Encouraging meaningful daily activities, which help maintain autonomy and purpose.

These interventions are especially useful in the context of chronic pain and fatigue, which are common in PPG and can further contribute to emotional exhaustion (17).

Regular screening for psychological distress using validated tools (e.g., PHQ-9, GAD-7) may facilitate early detection and referral. Nurses should document behavioral changes (e.g., withdrawal, tearfulness, frustration) and collaborate with physicians or mental health professionals to ensure timely intervention.

### 5.4 Nutritional support

Nutrition is a critical yet often under-recognized component of wound healing, particularly in patients with chronic inflammatory conditions such as peristomal pyoderma gangrenosum (PPG). Malnutrition impairs immune function, delays collagen synthesis, reduces angiogenesis, and increases susceptibility to infection—all of which contribute to delayed wound healing and prolonged hospital stays. Given the catabolic burden imposed by chronic inflammation and ongoing tissue repair, early nutritional assessment and intervention are essential.

#### 5.4.1 Nutritional assessment

Systematic screening for malnutrition risk should be initiated upon diagnosis of PPG and repeated throughout the treatment course, especially in patients with coexisting inflammatory bowel disease (IBD), advanced age, or significant wound exudation.

Tools such as the Nutritional Risk Screening 2002 (NRS-2002) are widely used in clinical settings to stratify patients. An NRS-2002 score of ≥3 indicates a high nutritional risk and necessitates immediate intervention under the guidance of a registered dietitian (36). Additional indicators include unintentional weight loss, hypoalbuminemia, muscle wasting, poor oral intake, and elevated inflammatory markers (e.g., CRP), which may signal increased metabolic demands.

Nurses play a frontline role in identifying patients at risk, coordinating dietitian referrals, and reinforcing individualized dietary plans during inpatient and outpatient care.

#### 5.4.2 Nutritional interventions

Once nutritional risk is identified, targeted interventions should address the three pillars of nutritional support in wound care: macronutrients, micronutrients, and hydration.

##### 5.4.2.1 Macronutrients

Adequate protein intake is the most important nutritional determinant of effective wound healing, as it supports collagen synthesis, immune cell function, and angiogenesis. High-protein diets (typically 1.2–1.5 g/kg/day) are recommended, with further adjustments for patients with large exudative wounds or muscle wasting. Caloric needs must also be met to spare protein for tissue repair rather than energy production. For patients unable to meet nutritional goals through diet alone, oral nutritional supplements (ONS) or enteral feeding may be required.

##### 5.4.2.2 Micronutrients

Wound healing demands are also increased for key vitamins and trace elements. Specific nutrients of importance include:

Vitamin C—promotes collagen cross-linking, antioxidant protection, and leukocyte activity.
Vitamin A and B-complex—support epithelialization, energy metabolism, and immune response.
Zinc—essential for DNA synthesis, fibroblast proliferation, and keratinocyte migration.
Iron—necessary for oxygen transport and collagen hydroxylation.

Deficiencies in these nutrients are common in patients with IBD or those on long-term corticosteroids. Supplementation should be based on laboratory testing and dietary intake assessment.

##### 5.4.2.3 Hydration

Adequate fluid intake is essential to maintain tissue perfusion, nutrient delivery, and metabolic clearance. Dehydration can impair wound healing by reducing cellular activity and delaying granulation. Patients with high-output stomas, diarrhea, or febrile illnesses are especially vulnerable to fluid deficits and may require monitoring of electrolyte balance and tailored hydration plans (12).

#### 5.4.3 Nutritional education

In addition to clinical interventions, patient education plays a vital role in sustaining nutritional improvements. Nurses and dietitians should provide individualized counseling on: high-protein food sources and meal planning, managing appetite changes or dietary restrictions (e.g., IBD flare adaptations), the role of supplements and safe use, reading food labels and portion control, the impact of nutrition on wound healing and recurrence prevention, empowering patients with practical nutritional knowledge fosters autonomy, improves adherence, and supports long-term wound stability.

### 5.5 Follow-up and health education

Ongoing follow-up and structured health education are essential components of long-term PPG management. Even after initial healing, patients remain at risk for recurrence, complications, and psychosocial distress, particularly if underlying inflammatory diseases (e.g., IBD) remain active or if ostomy care is suboptimal. A proactive follow-up strategy combined with tailored educational interventions, supports early detection of recurrence, enhances patient engagement, and promotes sustainable self-management.

#### 5.5.1 Regular assessments

PPG is characterized by a relapsing–remitting course, with recurrence reported in up to 61% of cases, particularly when systemic inflammation remains uncontrolled or mechanical irritation persists (27). Scheduled outpatient follow-up allows clinicians and nurses to assess: ulcer recurrence or new lesion formation, ongoing wound healing status (edge progression, granulation, epithelialization), patient-reported pain or changes in symptom burden, adherence to systemic and topical therapies, functionality of the stoma and peristomal skin condition.

Follow-up intervals should be individualized based on disease severity, wound size, comorbidities, and social support. High-risk patients may require weekly to biweekly follow-ups, transitioning to monthly once stability is achieved. During each visit, wound reassessment, pain monitoring, medication reconciliation, and psychosocial screening should be systematically performed.

Remote follow-up options, including telephone calls, mobile applications, or video consultations, can supplement in-person visits, especially for patients with mobility limitations or those living in rural areas. Digital wound photography, when feasible, enables clinicians to compare progress across visits.

#### 5.5.2 Health education and self-management training

Empowering patients with practical and personalized knowledge significantly enhances adherence and confidence in self-care. Tailored educational materials—such as printed guides, mobile apps, instructional videos, and interactive e-learning platforms—can improve retention and accessibility.

Key topics include:

Understanding the pathophysiology of PPG and the importance of avoiding trauma (pathergy).
How to identify early signs of recurrence (e.g., erythema, pain flare, pouch leakage).
Step-by-step guidance on wound care and appliance maintenance.
When and how to seek medical attention for changes.
Medication adherence and understanding the side effects of systemic therapy.

Self-management training should begin during hospitalization and continue through outpatient care. Techniques such as teach-back, return demonstration, and visual aids can help ensure comprehension (37). Caregivers should also be included when possible, especially if the patient has physical or cognitive limitations.

#### 5.5.3 Supportive interventions and holistic planning

Effective follow-up includes more than clinical checks; it requires addressing the broader logistical, emotional, and socioeconomic needs of patients. Many individuals face barriers such as: high cost of wound care products, stoma appliances, or systemic medications, transportation difficulties for frequent appointments, loss of income due to prolonged disability, caregiver burnout, or lack of support.

Multidisciplinary teams—including medical social workers, home health services, dietitians, mental health professionals, and wound care nurses—should work collaboratively to identify and mitigate these barriers. Referral to financial aid programs, community-based wound care resources, or peer support networks can significantly improve care access and continuity.

From a nursing perspective, creating personalized discharge plans, scheduling follow-up visits in advance, and providing direct contact points for questions can bridge the gap between hospital and home care. Such proactive strategies are essential for reducing readmissions, preventing avoidable deterioration, and supporting patient independence. Table 2 summarizes the key nursing strategies for PPG management across five domains—providing a structured reference for clinical practice and patient-centered care.

**Table 2:** Nursing strategies for peristomal pyoderma gangrenosum (PPG).

Nursing focus	Key actions	Detailed strategies and objectives	References
Wound care	Assessment	Regularly assess wound severity using scales like Bates-Jensen Wound Assessment Scale, focusing on wound size, depth, and exudate characteristics	Baranoski et al. (17)
	Dressing selection	Apply absorbent dressings (e.g., alginates, hydrocolloids) to maintain a clean, moist wound environment	Xu et al. (25)
	Ostomy appliance management	Evaluate and educate on stoma bag application techniques to minimize leakage and mechanical irritation	Hanley et al. (8)
Pain management	Comprehensive pain assessment	Use tools like Numeric Rating Scale (NRS) or Faces Pain Scale-Revised (FPS-R) to document patient pain levels comprehensively.	Wang et al. (36)
	Multidisciplinary management	Collaborate with physicians for personalized pain management plans, including pharmacologic and non-pharmacologic interventions	Wang et al. (36)
Psychological support	Counseling and emotional support	Provide resources such as support groups and personalized psychological counseling to alleviate anxiety and depression caused by chronic conditions	Guo et al. (18)
	Financial guidance	Help patients access resources to manage the financial burden of long-term care, including insurance options	Toyoda et al. (26)
Nutritional support	Counseling and emotional support	Provide high-protein, vitamin C, and zinc-rich diets to promote wound healing and reduce recurrence risks	Ding et al. (38)
	Fluid intake monitoring	Ensure adequate hydration to support metabolic processes necessary for recovery	Toyoda et al. (26)
Follow-up care	Regular monitoring	Conduct follow-up visits to detect early signs of recurrence, evaluate treatment efficacy, and adjust care plans accordingly	Becker et al. (27)
	Education	Develop tailored educational materials and training sessions to improve patients’ self-management abilities and adherence to prescribed treatments	Tsujinaka et al. (37)

A structured summary of nursing interventions for PPG, including wound care, pain management, psychological support, nutritional counseling, and follow-up planning. Each category outlines key actions and detailed strategies, with supporting literature to guide evidence-based practice and multidisciplinary coordination.

## 6 Discussion

Peristomal pyoderma gangrenosum (PPG) is an uncommon yet severe dermatologic complication typically associated with stoma formation, often in the context of inflammatory bowel disease (IBD). Despite advances in medical and surgical care, the diagnosis and management of PPG remain significant clinical challenges. This review synthesizes current evidence on the etiology, diagnosis, multidisciplinary treatment, and nursing management of PPG and proposes integrated frameworks for both therapeutic and nursing care. However, substantial gaps in standardization and clinical evidence remain.

### 6.1 Clinical complexity and diagnostic delay

One of the principal challenges in managing PPG lies in its nonspecific clinical presentation and overlapping features with other peristomal complications. Misdiagnosis as irritant contact dermatitis, surgical site infection, or fungal disease is common, contributing to treatment delay and worsening ulceration (1–5). Although several diagnostic frameworks have been proposed, including those by Su et al. (13) and the Delphi (14) consensus statements, none have yet achieved universal acceptance. Biopsy remains an essential, though nonspecific, tool in excluding mimickers. The need for high clinical suspicion and multidisciplinary collaboration is thus a recurring theme in the literature.

Emerging digital wound imaging, AI-assisted diagnostics, and three-dimensional technologies hold promise for improving early diagnosis and real-time monitoring, particularly in institutions with robust wound care infrastructure. However, the adoption of these tools remains limited by access, cost, and lack of validation in PPG-specific populations.

### 6.2 Evolving therapeutic strategies: balancing efficacy and safety

Management of PPG requires a delicate balance between controlling local ulceration and suppressing systemic immune dysregulation. Local wound therapies remain the first-line interventions, especially in mild or early-stage PPG. Techniques such as enzymatic debridement, NPWT (vacuum-assisted closure), and hydrocolloid dressings are essential for preparing the wound bed and controlling infection (12, 25). However, pathergy—lesion exacerbation following mechanical trauma—necessitates particular caution in the selection and application of local therapies.

Systemic therapeutic strategies have evolved significantly, with immunosuppressants and biologics taking center stage. Corticosteroids remain the mainstay of acute control, while calcineurin inhibitors, methotrexate, and mycophenolate mofetil are often used for steroid-sparing purposes (15, 16). More recently, targeted biologics—including TNF-α inhibitors (e.g., infliximab, adalimumab) and IL-12/23 inhibitors (e.g., ustekinumab)—have shown success in PPG, especially in patients with concurrent IBD (23, 24). Although these agents offer improved control and reduce the risk of pathergy, their cost, immunosuppressive risks, and limited long-term data remain constraints. To date, no head-to-head trials exist to guide optimal biologic selection.

A growing interest in traditional Chinese medicine (TCM) has also emerged, with several case reports and small cohort studies suggesting benefits in wound healing and systemic immune regulation (26). Nonetheless, TCM suffers from challenges of standardization, reproducibility, and integration into evidence-based protocols.

### 6.3 Role and timing of surgical intervention

Surgical therapy has traditionally been avoided in PPG due to concerns over pathergy and recurrence. However, selected patients with non-healing, large, or deep ulcers may benefit from surgical debridement, flap transplantation, and stoma revision or closure (6, 27). Multidisciplinary surgical planning—with preoperative immunosuppression and postoperative wound management—has emerged as a safe and effective strategy in high-volume centers.

Yet, surgical treatment remains controversial. Timing is critical: early surgery in an immunologically unstable patient may worsen the lesion, whereas delayed surgery risks progression and systemic infection. Future studies should aim to define optimal windows for surgery and preoperative preparation protocols.

### 6.4 The central role of nursing in integrated PPG care

This review highlights the indispensable contribution of nursing care in PPG management. Beyond supportive functions, specialized nursing interventions are central to wound care, pain control, patient education, and long-term monitoring. Wound care nurses are responsible for dressing changes, exudate assessment, and preventing ostomy-related complications. Pain management, particularly through the integration of pharmacological and non-pharmacological approaches, reduces suffering and improves compliance.

Psychological support, including counseling and group therapy, addresses anxiety, depression, and body image concerns. Nutrition and hydration monitoring, especially in patients with IBD and chronic wounds, improves healing outcomes and reduces recurrence. Follow-up care and patient education foster long-term self-efficacy, which is essential given recurrence rates as high as 60%. Tables 1, 2 provide structured frameworks for integrating medical and nursing strategies. However, most evidence supporting nursing interventions remains limited to Level III or below (observational studies, expert opinion), underscoring the need for nurse-led clinical trials and guideline development in this field.

### 6.5 Knowledge gaps and research opportunities

Despite decades of accumulated clinical experience, PPG remains an under-researched domain. Specific gaps include: (1) a lack of randomized controlled trials comparing systemic therapies, particularly biologics. (2) Uncertainty in the diagnostic criteria, especially for early-stage or atypical PPG. (3) Limited understanding of the pathophysiology, particularly the role of neutrophilic dysfunction, genetic susceptibility, and the microbiome. (4) Underrepresentation of nursing strategies in clinical research and guidelines.

Minimal data on cost-effectiveness and access disparities in PPG care, particularly in rural or low-resource settings. Future research should aim to: (1) Validate biomarkers for disease onset, activity, and response to treatment. (2) Develop and test integrated care pathways, including digital tools, AI wound monitoring, and standardized nursing protocols. (3) Promote interprofessional collaboration, including nurse-led research, to define best practices in long-term management.

## 7 Conclusion

Peristomal pyoderma gangrenosum (PPG) is a complex condition that requires early recognition, appropriately aggressive yet individualized treatment, and sustained multidisciplinary collaboration. Integrating medical, surgical, and nursing strategies remains the cornerstone of optimal PPG care. As understanding of immune mechanisms and wound biology continues to evolve, approaches to patient-centered and evidence-based interventions must evolve accordingly. Continued research and innovation, particularly involving the nursing workforce, are essential for improving outcomes in this rare but life-altering condition.
